# In-vivo evaluation of vitamin E loaded muscle void fillers for the provisional treatment of volumetric muscle loss

**DOI:** 10.1186/s13104-025-07454-2

**Published:** 2025-08-29

**Authors:** Michael S. Valerio, Andrew R. Clark, Marissa N. Behun, Christopher L. Dearth, Stephen M. Goldman

**Affiliations:** 1 Extremity Trauma and Amputation Center of Excellence, Defense Health Agency, Falls Church, VA USA; 2 Department of Surgery, Uniformed Services University of the Health Sciences, Bethesda, MD USA; 3 The Henry M. Jackson Foundation for the Advancement of Military Medicine, Inc, Bethesda, MD 20814 USA

**Keywords:** Orthopaedics, Trauma, Orthobiologics, Biomaterials, Military personnel

## Abstract

**Objective:**

This study investigates the therapeutic potential of α-tocopherol (Vitamin E, VitE), delivered locally via a polyethylene glycol (PEG) hydrogel serving as a muscle void filler (MVF), for the acute treatment of volumetric muscle loss (VML) in a rat model. The primary goal was to determine if VitE, a reactive oxygen species scavenger, could exert antioxidant effects at the VML site and thereby improve the recovery of adjacent muscle tissue over a four-week period.

**Results:**

The MVF successfully increased VitE levels in the muscle throughout the implantation period. However, only the lowest concentration of VitE (5 mg/ml) showed a demonstrable effect, resulting in a 30% reduction in peak force compared to the PEG-alone group and a median muscle fiber size that was 4.34 μm smaller. Biochemical analysis revealed no differences in the expression of TGFβ1, protein carbonyl, or malondialdehyde across any groups. While local delivery of VitE was successful, further research is needed to optimize dosing and investigate additional mechanisms for improved muscle recovery.

## Introduction

Volumetric muscle loss (VML), resulting from traumatic injuries or surgery, leads to significant functional deficits and impaired quality of life due to the limited capacity for muscle fiber regeneration [1]. Previously, we have investigated the use of synthetic polymer-based muscle void fillers (MVF) to stabilize the VML injury for delayed treatment [2, 3]. While these early MVFs successfully preserved the defect volume, they didn’t improve the existing musculature during the acute recovery period. To address this, we now aim to leverage the MVF as a versatile platform for delivering bioactive payloads. In this study, we investigate the therapeutic potential of locally delivering α-tocopherol (Vitamin E, VitE), using a polyethylene glycol (PEG) hydrogel as the MVF delivery vehicle. As a known reactive oxygen species (ROS) scavenger [4, 5], VitE has shown promise in mitigating muscle injury and aiding membrane repair [6–10]. As such we hypothesized that VitE delivered via the MVF would exert antioxidant effects at the VML site, promoting recovery of surviving muscle and priming the injury for subsequent definitive regenerative therapy.

## Materials and methods

### Animal care and use

This study was approved by our local Institutional Animal Care and Use Committee and conducted in accordance with the Animal Welfare Act and the principles outlined in the Guide for the Care and Use of Laboratory Animals [11]. Animals were purchased from Inotiv, Inc. (Indianapolis, IN), housed in the AAALAC-accredited facilities of the Uniformed Services University of the Health Sciences, maintained on a 12-hour light/dark cycle, and provided with ad libitum access to food and water. To minimize variability, and reflect the predominantly male active-duty demographic, the study was limited to male subjects.

### Experimental design and injury model

An established VML injury procedure [12] was performed on adult male Sprague-Dawley rats weighing approximately 300–350 g. Following anesthesia induction with isoflurane (5%) and maintenance (1–3%), and administration of preoperative analgesia (buprenorphine ER, 1.2 mg/kg, Wedgewood Pharmacy), a full-thickness biopsy of the tibialis anterior (TA) muscle was created using a 6 mm punch. Animals were subsequently block-randomized into four treatment groups (*n* = 10/group): a PEG-only control and groups receiving PEG loaded with VitE at concentrations of 5, 25, or 50 mg/mL. To generate the MVF, approximately 100 µL of a sterile solution containing 7% w/v of 10 kDa 8-arm PEG prepolymer in 0.9% saline, along with 10 µg/mL Irgacure (CAT# 410896, MilliporeSigma), was prepared with or without the specified concentrations of VitE. This solution was pipetted into the VML defect and polymerized into a hydrogel via 1 min of ultraviolet (UV) light exposure (385 nm; 6,800 mW/cm²). The fascia and skin were then closed with sutures. Animals were subsequently euthanized while under a surgical plane of isoflurane anesthesia via intracardiac injections of a lethal dose (0.5mL) of pentobarbital sodium and phenytoin sodium solution (EUTHASOL^®^, Virbac Corporation, Westlake, TX) at 7 and 28 days post-operatively for assessment of study outcomes. At each endpoint, anterior crural muscles were harvested, weighed (wet), and randomly allocated to biochemical (*n* = 5/group/endpoint) or histological (*n* = 5/group/endpoint) outcomes.

### Neuromuscular strength and functional assessment

In vivo isometric torque was measured using a dual-model muscle lever system (Model#305 C-LR; Aurora Scientific, Aurora, Canada). Anesthetized rats (1–2% isoflurane) were stimulated via transcutaneous needle electrodes placed on either side of the common peroneal nerve. Stimulation voltages were optimized for each animal using twitch and tetanic contractions (150 Hz, 0.1 ms pulse width, 400 ms train). To isolate TA-specific torque, a surgical window was opened at the antero-lateral aspect of the ankle to expose and sever synergist muscles. Isometric tetanic torque from the TA muscles was then measured between 10 and 200 Hz with the ankle fixed at 90°.

### Biochemical assays

Whole snap-frozen TA and extensor digitorum longus (EDL) muscles were crushed to powder using a mortar and pestle with liquid nitrogen and homogenized in TPER and HALT solution. Samples were centrifuged, and the supernatant was collected. Total protein concentration was quantified using a Pierce TM BCA Protein Assay Kit (Thermo Fisher Scientific Inc.; Waltham, MA, USA) according to the manufacturer’s instructions. VitE, TGFβ1, Protein Carbonyl (PC), and Malondialdehyde (MDA) were measured by ELISA from protein extracted at Day 7 to assess the bioavailability of VitE and its impact on oxidative stress, antioxidant defense, and fibrotic signaling in the local wound environment during the subacute inflammatory phase of tissue repair. Myosin Heavy Chain (MyHC) isoforms: MYH1 (MyHC-IIx), MYH2 (MyHC-IIa), and MYH3 (MyHC-embryonic) were measured by ELISA from protein extracted at Day 28 to provide critical insights into the formation of new muscle fibers, their maturation, and the progression towards functional recovery during the later stages of skeletal muscle regeneration and remodeling. All ELISA kits were purchased from MyBioSource, Inc., and assays were performed meticulously following the manufacturer’s protocols, using their provided diluents and reagents to ensure accuracy and consistency.

### Histology and immunohistochemistry

Muscles were fixed in 10% formalin, cryoprotected with a sucrose gradient, and snap-frozen in optimal cutting temperature (OCT) compound using liquid-nitrogen-cooled isopentane. 5 μm sections were prepared (Histoserv, Inc., Germantown, MD) and stained with hematoxylin and eosin (H&E) for general pathology, picrosirus red staining (PSR) for collagen content, and fluorescently labeled Wheat Germ Agglutinin (WGA) for myofiber morphometrics. PSR staining was performed using a commercial kit (Abcam; Cambridge, United Kingdom) according to the manufacturer’s protocol. For WGA staining, sections were stained with WGA-AF488 (Thermo Fisher Scientific Inc.; Waltham, MA, USA) and DAPI (1:2000, MilliporeSigma), then mounted with glycerol/PBS (1:1). Slides were imaged using a Zeiss Axio Scan Z1 (Zeiss; Oberkochen, Germany) at 20X (PSR) and 10X (WGA) with standardized parameters. Quantitation was performed using the HALO Image analysis platform (Indica Labs, New Mexico, USA). For PSR analysis, five muscle cross-sections from the defect region were analyzed to determine the percentage of total tissue area. For WGA analysis, fiber count and size were collected from entire tissue cross-sections. Fiber size was defined as the minimum diameter from 12 measurements within each fiber. Individual object data were then imported into RStudio for distribution analysis.

### Statistical analysis

All datasets were subject to an outlier test prior to analysis to remove likely outliers. To compare the effect of hydrogels with varying concentrations of VitE to control, data were analyzed using One-Way ANOVA and Dunnett’s post hoc tests. Comparison between time points were compared using a Two-way ANOVA with Dunnett’s post hoc test. For frequency and distribution analysis, a mixed-effects Two-way ANOVA with Tukey’s multiple comparison post hoc tests were performed. Results are presented as mean ± standard deviation.

## Results

### Effect of vitE hydrogels on gross anatomy and neuromuscular function

**Fig. 1:**
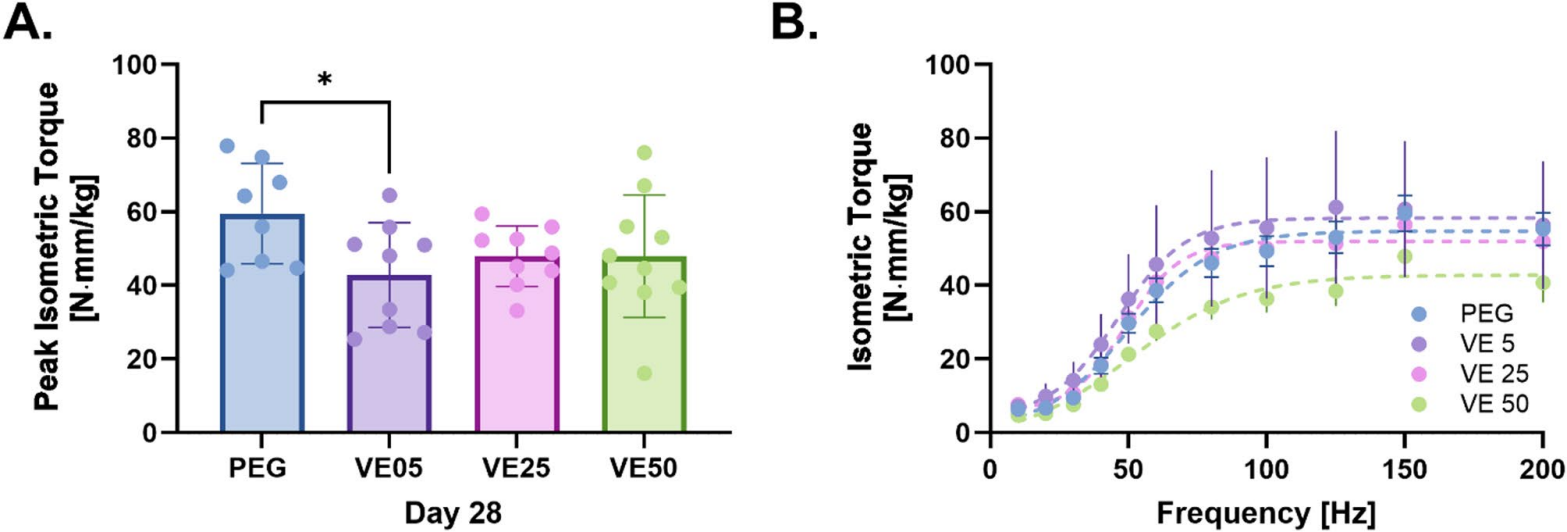
Neuromuscular function. (**a**) Peak isometric torque of dorsiflexion following peroneal nerve stimulation. One-Way ANOVA and Dunnett’s post hoc test (**b**) Force-frequency response of dorsiflexion torque. Mixed-effects Two-way ANOVA. *P-value < 0.05

The wet weight of the tissue removed during the surgical creation of the VML injury (i.e. defect weight, DW) for all groups ranged 97.1-107.3 mg with no differences between groups (Table 1). Body weight (BW) at surgery revealed that VitE5 and VitE50 were heavier than PEG and VitE25 at both Day 7 (*P* < 0.05) and Day 28 (*P* < 0.05). At Day 7, VitE5 and VitE50 groups gained weight, unlike PEG and VitE25, which lost weight (*P* < 0.05). While at Day 28, only VitE25 weighed less than PEG (*P* < 0.05), the overall weight change showed that PEG and VitE25 gained more weight than VitE5 and VitE50 (*P* < 0.05), with differences in body weight between endpoints (*P* < 0.05). Uninjured raw TA/EDL muscle weights (MW) were not different compared to PEG at Day 7 or 28. Injured raw TA/EDL MW were also not different at Day 7 compared to PEG but at Day 28 VitE50 was heavier than PEG (*P* < 0.05). When normalized to BW, at Day 28, VitE5 was slightly larger (*P* < 0.05) compared to PEG in uninjured limb and VitE50 was larger (*P* < 0.05) than PEG in the injured limb. In vivo neuromuscular function results evaluating peak isometric torque and torque frequency data are highlighted in Fig. 1. VitE5 treated animals had a near 30% reduction in peak force compared to PEG alone (*P* < 0.05). Comparing force frequencies using a mixed-effects analysis, results reveal no differences in force between groups at each frequency.

**Table 1:** Gross anatomy and muscle weights

Day 7 Outcomes	PEG	VitE5	VitE25	VitE50
*Defect Weight (mg)*	100.5 ± 10.8	98.3 ± 14.4	107.3 ± 14.8	97.1 ± 12.7
*Surgery Body Weight (g)*	336.3 ± 15.6	356.8 ± 14.6*	332.3 ± 7.8	353.5 ± 8.8*
*Endpoint Body Weight (g)*	331.7 ± 12.5	360.6 ± 13.9*	332.2 ± 9.0	356.9 ± 9.9*
*Change in BW (g)*	-4.60 ± 9.17	3.80 ± 3.55*	-0.10 ± 6.32	3.40 ± 4.99*
*Injured Dorsiflexor Wet Weight (g)*	0.946 ± 0.79	0.989 ± 0.08	0.906 ± 0.09	1.02 ± 0.06
*Contralateral Dorsiflexor Wet Weight (g)*	0.83 ± 0.04	0.84 ± 0.4	0.79 ± 0.07*	0.83 ± 0.4
*Normalized Wet Weight of* *Contralateral Dorsiflexor [mg/g]*	2.50 ± 0.14	2.33 ± 0.9*	2.39 ± 0.21*	2.34 ± 0.13*
*BW Normalized Wet Weight of* *Injured Dorsiflexor Muscles [mg/g]*	2.86 ± 0.31	2.88 ± 0.45	2.73 ± 0.31	2.81 ± 0.19
$\:\frac{Injured\:Dorsiflexor\:Wet\:Weight\:\left(g\right)}{Contralateral\:Dorsiflexor\:Wet\:Weight\:\left(g\right)}$	1.145 ± 0.11	1.24 ± 0.19	1.15 ± 0.11	1.21 ± 0.1
**Day 28 Outcomes**	PEG	VitE5	VitE25	VitE50
*Defect Weight (mg)*	104.2 ± 12.1	106 ± 14.4	103.4 ± 13.5	103.6 ± 9
*Surgery Body Weight (g)*	334.6 ± 23.5	365.1 ± 8.1*	328.8 ± 9.8	364.2 ± 15.4*
*Endpoint Body Weight (g)*	402.1 ± 8	415.2 ± 8.3	383.1 ± 18.4*	414 ± 19.6
*Change in BW (g)*	67.5 ± 20.3	47.5 ± 10.4*	54.3 ± 13.3*	49.8 ± 10.2*
*Injured Dorsiflexor Wet Weight (g)*	1.1 ± 0.1	1.19 ± 0.1	1.09 ± 0.1	1.27 ± 0.07
*Contralateral Dorsiflexor Wet Weight (g)*	1.02 ± 0.07	1.09 ± 0.06*	0.96 ± 0.08	1.05 ± 0.09
*Normalized Wet Weight of* *Contralateral Dorsiflexor [mg/kg]*	2.54 ± 0.18	2.65 ± 0.14*	2.51 ± 0.17	2.52 ± 0.13
*BW Normalized Wet Weight of* *Injured Dorsiflexor Muscles [mg/kg]*	2.73 ± 0.22	2.91 ± 0.26	2.85 ± 0.22	3.00 ± 0.19
$\:\frac{Injured\:Dorsiflexor\:Wet\:Weight\:\left(g\right)}{Contralateral\:Dorsiflexor\:Wet\:Weight\:\left(g\right)}$	1.08 ± 0.13	1.1 ± 0.1	1.14 ± 0.1	1.19 ± 0.08

Notes: All data are Mean ± SD. *P < 0.05 relative to PEG at same timepoint

### Muscle fiber and fibrosis quantification

**Fig. 2:**
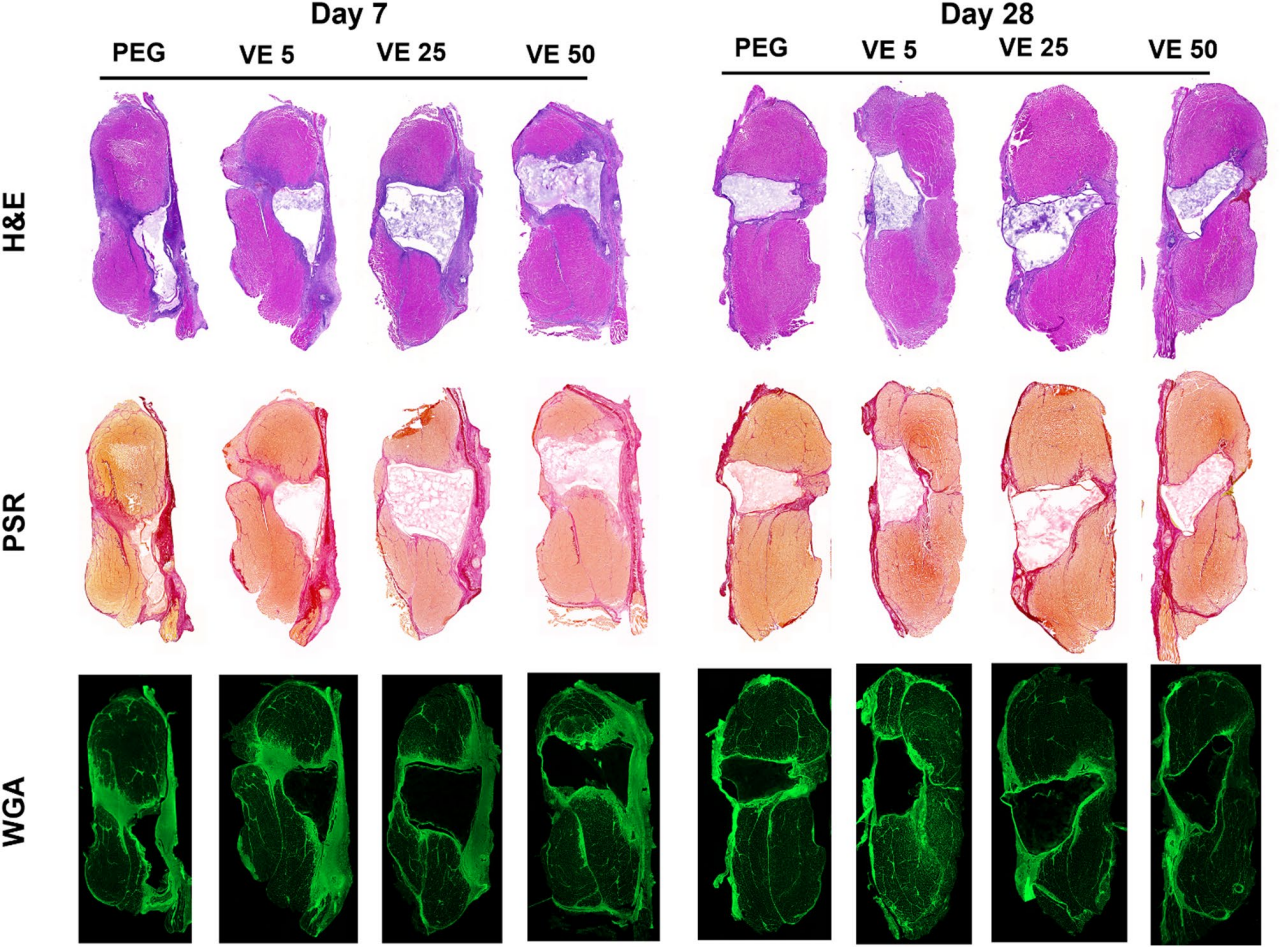
Representative H&E PSR and WGA stained histological cross sections. Quantification of the PSR stained sections showing (**a**) total collagen area and (**b**) percentage of the cross-sectional area that is collagen. WGA sections were analyzed to determine (**c**) number of fibers in each histological cross section, (**d**) total area of muscle tissue, (**e**) median fiber size, (**f**) kernel density distribution of fiber size

Histological analyses of PSR and WGA-stained tissue sections were performed to assess the effect of VitE-loaded hydrogels on tissue fibrosis and myofiber regeneration (Fig. 2). Muscle fibrosis showed no differences in total fibrotic area or fibrotic tissue fraction across treatment groups compared to control at either Day 7 or Day 28 post-injury (Table 2). Evaluation of muscle fiber size via WGA staining revealed limited differences between treatment groups. Absolute fiber count did not differ from the PEG control at either Day 7 or Day 28 (Table 2). Total muscle tissue area was slightly increased in the VitE5 treatment group compared to control. Median fiber area, however, showed a slight decrease in the VitE5 group compared to the PEG control (*P* < 0.05). Day 7 results showed no differences in mean or distribution among treatment groups compared to control. At Day 28, a slight decrease in fiber distribution was observed in the VitE5 group (Table 2).

**Table 2:** Histomorphometric outcomes

DAY 7 OUTCOMES	PEG	VitE5	VitE25	VitE50
*Cross-Sectional Area [mm* ^*2*^ *]*	23.4 ± 10.0	32.2 ± 4.2*	27.7 ± 4.3	26.6 ± 4.6
*% Fibrotic Area*	34.6 ± 10.4	28.5 ± 10.1	24.0 ± 6.3	34.5 ± 9.6
*Myofibers (n)*	8.16e3 ± 4.35e3	1.10e4 ± 1.81e3	8.41e3 ± 1.80e2	8.79e3 ± 1.51e3
*Median Min. Feret Diameter (µm)*	29.2 ± 2.4	30.1 ± 1.9	31.0 ± 2.0	30.6 ± 1.4
DAY 28 OUTCOMES	**PEG**	**VitE5**	**VitE25**	**VitE50**
*Cross-Sectional Area [mm* ^*2*^ *]*	38.1 ± 3.4	36.3 ± 6.0	32.7 ± 2.5	40.6 ± 4.2
*% Fibrotic Area*	21.7 ± 9.6	36.1 ± 14.0	10.1 ± 9.0	24.9 ± 14.9
*Myofibers (n)*	1.02e4 ± 1.31e3	9.58e3 ± 2.34e3	8.00e3 ± 6.07e2	1.11e4 ± 9.55e2
*Median Min. Feret Diameter (µm)*	33.7 ± 4.5	29.3 ± 4.0*	35.9 ± 2.6	30.9 ± 5.8

Notes: all data are Mean ± SD *P < 0.05 relative to PEG at same timepoint

### Effect of vitE hydrogels on VML-induced oxidative stress, growth factors and myogenic protein expression

**Fig. 3:**
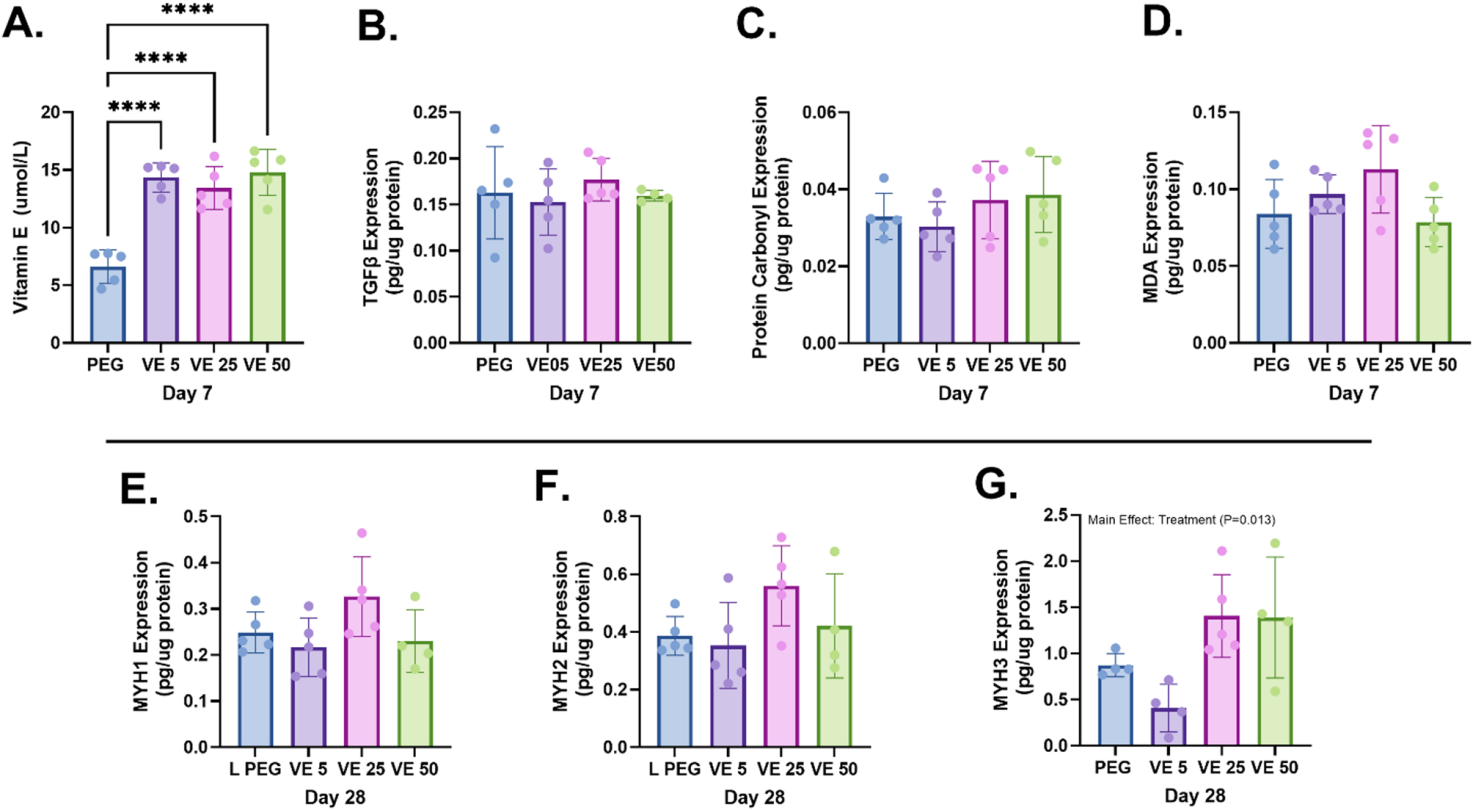
Biochemical assays. Quantification of (**a**) Vitamin E, (**b**) TGFβ, (**c**) protein carbonyl, (**d**) MDA 7 days after injury in the injured muscle. Protein quantification of (**e**) MYH1, (**f**) MYH2, and (**g**) MYH3 in the VML injured muscle 28 days after injury. One-Way ANOVA and Dunnett’s post hoc tests. ****P-values < 0.001

Protein expression from Day 7 treated muscles is shown in Fig. 3. VitE expression was measured in crushed muscles from each group and reveals that compared to PEG only, all VitE hydrogel treated tissues had more VitE (*P* < 0.001), although there were no differences between VitE groups. Looking at TGFβ1, the results show no differences in expression between groups. Moreover, expression of Protein Carbonyl and MDA were also not different among treatments vs. PEG control. Additionally, MYH1 and MYH2 expression were not different than PEG controls. MYH3 expression showed a main effect by treatment (*P* = 0.013) but no difference in any specific treatment compared to PEG control.

## Discussion

This study investigated the potential of local Vitamin E (VitE) delivery via a PEG hydrogel to acutely treat volumetric muscle loss (VML) in a rat model. While the hydrogel preparation successfully incorporated and delivered VitE to the tissues, the results present a complex picture that doesn’t fully support the initial hypothesis regarding VitE’s therapeutic benefit.

Biochemical analysis confirmed that local delivery of VitE successfully increase local bioavailability of the antioxidant within the VML wound bed. Despite its presence at the injury site, VitE did not modulate the expression of PC and MDA, which are markers of oxidative stress [13, 14]. Similarly, no differences were observed in the levels of TGFβ1, a key growth factor involved in fibrosis and muscle regeneration [15]. This suggests that, contrary to its known antioxidant properties, VitE did not significantly modulate inflammation and oxidative damage during the subacute inflammatory phase of VML wound repair.

Furthermore, analysis of myosin heavy chain (MYH) isoforms, which are indicators of fiber type shifting and regeneration, provided some insights into the regenerative response [16, 17]. While no differences were observed in MYH1 and MYH2 expression (markers of mature fiber types) between treatment groups and the PEG control, MYH3 (embryonic myosin), a key marker of regenerating fibers [18], showed a main effect of treatment. Specifically, the PEG, VitE25, and VitE50 groups all exhibited upregulation of MYH3 expression when normalized to the contralateral limb. This suggests that the hydrogel itself, with or without VitE, might be promoting some level of regenerative activity. However, the lack of a clear dose-dependent effect of VitE on MYH3 upregulation is notable, challenging the idea that increasing VitE concentration would proportionally enhance regeneration.

While the upregulation of MYH3 at day 28 suggested some initial regenerative activity across treatment groups, including those receiving VitE, the absence of corresponding improvements in total muscle tissue area at this later time point, coupled with the observed decrease in fiber size in the VitE5 group, paints a more nuanced and ultimately disappointing picture. The quantitative histological findings indicate that any early regenerative cues, potentially initiated by the hydrogel or low-level VitE activity, did not translate into sustained and meaningful muscle growth or fiber maturation. This lack of architectural improvement, particularly the negative impact on fiber size at the lowest VitE concentration, strongly aligns with the overall failure to observe functional benefits, reinforcing the conclusion that VitE, at the concentrations and delivery method tested, was not an effective therapeutic for VML in this model.

Interestingly, animals in the lowest and highest VitE groups did exhibit higher body weights. This unexpected observation warrants further investigation to understand the potential systemic effects of localized VitE delivery at these concentrations and to ensure the safety and translatability of this approach.

In summation, while the local delivery of VitE via a PEG hydrogel was achieved, the results of this study do not provide strong evidence for a significant therapeutic benefit of inclusion of VitE. The lowest concentration of VitE appeared to negatively impact muscle function, while higher concentrations did not improve functional outcomes, fibrosis, or markers of oxidative stress and regeneration at the measured time points. Further investigation into the temporal dynamics of VitE release and its effects on a broader range of molecular and cellular processes is warranted to fully elucidate its potential role in VML treatment. The observed systemic effects on body weight in the higher VitE groups also necessitate further study to ensure the safety and translatability of this approach.

### Limitations

A few limitations of this study warrant consideration. First, while the animal model effectively simulates VML in males, its direct translatability to the complex and heterogeneous nature of human VML injuries in both biological sexes may be limited. Second, the study focused on relatively early time points (7 and 28 days), and longer-term effects of VitE treatment on muscle regeneration and functional recovery remain to be elucidated. Third, the study assessed only a limited number of molecular markers; a more comprehensive analysis of the inflammatory and regenerative processes could provide a more complete understanding of the effects of VitE. Fourth, the study lacked an untreated control group. This omission makes it difficult to definitively determine whether the MVF-VitE treatment was beneficial or detrimental compared to no treatment for VML. Finally, the observed increase in body weight in the higher VitE concentration groups suggests potential systemic effects that require further investigation.

## Data Availability

Data will be made available upon request.
